# Low-Temperature Plasma for Livestock and Poultry Preservation: Bactericidal Effects, Mechanisms and Application Progress

**DOI:** 10.3390/foods15203729

**Published:** 2026-10-20

**Authors:** Hui Wang, Lei Yin, Jiaming Chen, Zhongliang Sun, Zekun Chen, Jianxiong Hao, Xuechao Zheng

**Affiliations:** 1 School of Food Science & Biology, Hebei University of Science & Technology, Shijiazhuang 050018, China; 13517012316@163.com (H.W.); 15003246619@163.com (J.C.); hbkjdxczk@163.com (Z.C.); 2 Hebei Seed Station, Shijiazhuang 050031, China; lyinhebeiapqsi@163.com; 3 College of Life Science, Yantai University, Yantai 264006, China; zlsun@ytu.edu.cn

**Keywords:** low-temperature plasma, microbial inactivation, oxidative regulation, meat quality

## Abstract

Livestock and poultry meat is an essential nutrient source, but significant losses occur during circulation. This review focusing on the generation and classification of low-temperature plasma (LTP), its application effects in preserving different types of livestock and poultry, and the core mechanisms involving microbial inactivation, regulation of meat physicochemical properties, and improvement of sensory attributes. The influences of different discharge modes, gas media, and process parameters on preservation effects were analyzed. LTP realizes preservation via reactive oxygen/nitrogen species, which sterilize by damaging microbial cell membranes, and regulate lipid oxidation and protein conformation to maintain meat quality. Different discharge forms, parameters and excessive treatment significantly affect preservation effects. Future research should focus on molecular mechanisms, personalized preservation schemes, low-cost equipment and process standardization. Integrating LTP with other technologies and intelligent equipment will enhance its industrial application, reduce meat losses and ensure food safety, providing theoretical support for improving meat preservation.

## 1. Introduction

Livestock and poultry meat is an indispensable source of nutrients in the human diet, which can not only serve as the core provider of high-quality protein, but also promote nervous system development, maintain immune function through rich heme iron, sodium, zinc, selenium and vitamin B_12_, and provide essential fatty acids for the human body, occupying a central position in the human diet [1]. Edible livestock and poultry meat has rich sources, and can be divided into livestock meat (pigs, cattle, sheep, rabbits) and poultry meat (chickens, ducks, geese) according to the types [2]. However, with the growth of the global population and the improvement of living standards, the consumption of livestock and poultry meat has also continued to rise. According to the statistics of the Food and Agriculture Organization of the United Nations (FAO), global meat production has exceeded 350 million tons in 2023, of which about 15–20% is lost in the circulation process due to corruption and spoilage, causing huge economic losses and waste of resources. Livestock and poultry meat spoilage is mainly caused by microbial proliferation. Lipid oxidation and protein degradation can also cause the deterioration of meat quality [3]. Although traditional preservation technologies such as refrigeration, chemical preservatives and irradiation can delay deterioration, they have limitations such as high energy consumption, risk of chemical residues and nutritional damage [4].

Low-temperature plasma (LTP), as an emerging non-thermal processing technology, has shown unique advantages in the field of food preservation in recent years [5]. It produces a hybrid system containing reactive oxygen species (ROS), reactive nitrogen species (RNS), ultraviolet rays, and high-energy particles through ionized gas, which can achieve efficient sterilization, inhibit oxidation, and maintain food quality at room temperature [6]. Compared with traditional techniques, this method has the characteristics of no chemical residues, low energy consumption, and environmental friendliness [7]. At present, LTP has been studied and applied in the fields of fruits and vegetables, aquatic products and dairy products.

A number of existing reviews have discussed the application of LTP in food preservation, their core scopes diverge from the current investigation. For instance, Subrahmanyam, K. et al. [8] provided a thorough overview of cold-plasma-driven microbial inactivation in animal-derived foods (meat, eggs, and seafood included). Their work mainly focused on inactivation efficacy, relevant mechanisms covering ROS/RNS-triggered membrane damage and toxin breakdown, as well as how processing parameters influence quality traits including lipid oxidation and color. Nevertheless, their coverage of meat-oriented quality modulation remained relatively broad, without establishing systematic connections between discharge configurations and resultant preservation performance. Meanwhile, Bak, K.H. [9] reviewed the impacts of atmospheric-pressure cold plasma (ACP) on flavour and volatile profiles of meat and meat products. Representative ACP sources for meat processing cover DBD, plasma jets, in-package ACP, and cold filamentary microplasma. Sensory outputs show considerable variability, spanning desirable flavour enhancement to undesirable off-odour development, as conditioned by meat substrate, processing gas and operational parameters. Modifications of volatile constituents are dominated by aldehydes, alcohols, esters and ketones that derive largely from lipid oxidation. One major drawback pointed out in this review is the lack of quantitative detection for in-situ generated reactive species in most existing investigations. This deficiency impedes mechanistic elucidation of flavour variations, optimisation of plasma processing conditions, as well as the evaluation of compliance requirements under the EU novel food regulation.

The present review is confined to livestock and poultry meat. It traces how discharge mode and gas medium shape preservation outcomes, integrates microbial inactivation, oxidative changes and sensory quality into a unified discussion, and discusses combined treatments separately to distinguish their effects from those of LTP alone. What follows is an account of LTP research in livestock and poultry preservation, with attention to microbial control, lipid oxidation inhibition and meat quality maintenance. How discharge method, power level and gas composition affect shelf life, color and texture is discussed, together with the limitations that remain. The aim is to offer a practical reference for applying LTP to meat preservation.

This review was conducted through a systematic search of Web of Science, Scopus, and PubMed, covering the period from 2016 to 2026. Search terms combined technology-related keywords (“cold plasma,” “low-temperature plasma,” “dielectric barrier discharge,” “plasma-activated water”), matrix-related keywords (“meat,” “pork,” “beef,” “mutton,” “chicken,” “duck”), and outcome-related keywords (“preservation,” “microbial inactivation,” “lipid oxidation,” “protein oxidation,” “quality,” “texture,” “color,” “flavor,” “shelf life”). Studies were included if they were peer-reviewed, applied LTP to livestock or poultry meat, and reported at least one quantitative microbiological, physicochemical, or quality indicator. Conference abstracts, patents, and studies on non-meat matrices were excluded. A total of 88 references (including four classic articles from ten years ago) were ultimately included. Studies that reported LTP parameters alongside quantitative preservation outcomes were compiled in tables in this work for cross-study comparison, while those focusing on mechanisms, protein structure, or flavor metabolites were addressed in the main text.

## 2. Generation and Classification of Low-Temperature Plasma Technology

Plasma is a quaternary state of matter in addition to gaseous, liquid, and solid states [10]. When gas molecules gain sufficient energy (such as by ionization), their intermolecular interactions will be broken, forming a quasi-neutral ionized gas state containing active species such as positive and negative ions, electrons, free radicals, and ultraviolet photons. Based on temperature and degree of ionization, plasma is divided into thermal and non-thermal plasma, the latter also called LTP, it produced by applying an electric or electromagnetic field to a gas [11]. In the temperature range of 30 °C to 60 °C [12], by ionizing the gas, LTP can be formed under atmospheric pressure or vacuum conditions. The discharge forms of low-temperature plasma mainly include dielectric barrier discharge (DBD), corona discharge (CD), microwave discharge (MD), and radio frequency discharge (RF), which are summarized in Table 1.

### 2.1. Dielectric Barrier Discharge

DBD is a form of non-equilibrium gas discharge in which the system consists of two electrodes, the core feature of which is to insert at least one layer of insulating medium (such as ceramic, glass, or polymer) between the discharge electrodes, limiting the continuous growth of the discharge current through the presence of this medium, thereby avoiding arc discharge. The system can generate plasma by ionizing the working gas, and in this process a large amount of ROS and RNS is produced as the two electrodes are ionized [21]. The dielectric blocks the discharge to stably generate non-thermal plasma at atmospheric pressure, avoiding electrode corrosion or contamination issues. DBD is currently widely used in cold sterilization, such as microbial inactivation on the surface of meat, fruits and vegetables, antibacterial modification of packaging materials, pesticide residue degradation and toxins [22,23,24,25].

### 2.2. Corona Discharge

CD is a phenomenon in which local air molecules are ionized and produce luminous and audible gas discharges when the electric field strength on the surface of the conductor exceeds the air ionization threshold under the action of high voltage. It is a process in which an electric current flow from an electrode with a high potential into a neutral fluid (air, Ar, N_2_, O_2_, etc.) and forms a plasma region around the electrode through the ionized fluid. Compared to other plasma types that require vacuum conditions, CD allows for efficient ionization even at atmospheric pressure due to its high electric field gradient [26]. CD usually occurs at the tip, edge, or surface of the electrode, and due to the uneven distribution of the electric field, ionization only continues in a localized area and does not form an arc through the entire gap. It is often used for air purification, electrostatic dust removal, and food packaging sterilization, especially suitable for high-flow gas treatment and open scenarios [27,28].

### 2.3. Microwave Discharge

MD is a physical process that uses microwave energy (typically at a frequency of 300 MHz–300 GHz) to excite a gas to produce plasma. The principle is to accelerate free electrons in gas through microwave electromagnetic fields, causing them to collide with neutral molecules and trigger ionization to form high-energy plasma. Unlike traditional discharge methods, MD does not require electrodes, avoids electrode material contamination, and has high energy transfer efficiency, generating high-density and uniform plasma. It is mainly used in semiconductor precision etching, high-performance thin film deposition, etc., and has significant advantages in cutting-edge manufacturing and green technology.

### 2.4. Radio Frequency Discharge

RF discharge is a technique that uses RF electromagnetic waves to excite gases to ionize to form plasma. The principle is to accelerate electrons through a high-frequency electric field, causing them to collide with gas molecules to produce a continuous discharge that can be maintained without high pressure. Capacitive coupling (electrode contact) or inductive coupling (magnetic field excitation) are commonly used, the former applies an electric field through the electrode, and the latter uses a coil to generate an alternating magnetic field. RF discharge has become the core technology in the fields of microelectronics, new energy and analysis and testing due to its high efficiency and controllability.

## 3. Application of Low-Temperature Plasma in the Field of Regulating the Quality Meat

It is important to note that “LTP” as used in the literature covers several technologically distinct configurations, which differ in how reactive species reach the meat: (a) direct plasma treatment, in which gas-phase ROS/RNS interact with the meat surface, (b) plasma-activated water (PAW) or solutions, in which reactive species are first dissolved in a liquid medium, (c) plasma treatment of packaging materials or edible films, in which plasma modifies the polymer surface to enable subsequent release, (d) plasma modification of extracts and biopolymers, in which plasma acts on an intermediate material rather than on the meat itself, and (e) combined procedures, in which LTP is used together with antioxidants, ultrasound, or other technologies. Since these configurations differ in reactive-species lifetime, penetration depth, effective dose, and industrial feasibility, their preservation effects cannot be compared directly. Therefore, each study discussed below is explicitly labeled according to its treatment configuration.

### 3.1. Livestock

Common edible livestock are mainly pigs, cattle, and sheep, which meet human needs for protein, fur, and labor. As a newly-developed technology, LTP achieves meat preservation by generating bacteriologically active compounds. These compounds disrupt microbial cellular structures and metabolic processes, suppress enzymatic activity, and slow down lipid oxidation. As outlined in Table 2, research concerning livestock meat is reviewed below based on differing treatment setups. In a packaging-based approach, Ye et al. [29] combined electrospinning with DBD cold plasma and antibacterial nanofibers, wrapping fresh pork with a D@MSNs-P/PCL nanofiber membrane before DBD treatment, this inhibited microbial growth, suppressed lipid oxidation and color change, and extended shelf life by 2 days. In another packaging application, citrus pectin was first modified by LTP and then cross-linked with chitosan to prepare a composite aerogel absorbent pad for chilled pork [30], after 90 s of N_2_ plasma treatment, the aerogel swelling and adsorption rates increased by 61.65% and 28.33%, respectively. Considering the total volatile basic nitrogen (TVB-N) and pH value, it delayed pork spoilage and extended the shelf life by 2–3 days compared to the 7-day control group. In a combined procedure of argon-cooled plasma (Ar-CP) with active packaging, an Ar-CP-treated polybutylene succinate/thermoplastic tapioca starch (PBS/TPS) film coated with 80% Makwaen essential oil (ME), under optimal conditions (120 W, 15 s), it can extended the shelf life of packaged pork sausages to 72 h at 25 °C, compared with 36 h for the control, and reduced TBARS by 0.45 mg MDA/kg, effectively inhibiting lipid oxidation and microbial growth [31].

In the field of beef preservation, LTP also has many applications. In a packaging-based approach, Deng et al. [45] developed an LTP-treated whey protein-carboxymethyl chitosan (WPI-CMCS) composite coating (CPW) and studied its effect on the quality and metabolites of chilled beef, and by optimizing the CP treatment conditions (applied voltage of 40 V for a duration of 3 min), CPW formed a stable complex, and its particle size, zeta potential and fluorescence spectra showed that the intermolecular interaction was enhanced and the structural stability was improved. The combined application of coating and LTP significantly reduced the content of TVB-N (7.91 mg/100 g) in chilled beef on the 5th storage days compared with the control group, delaying the discoloration of the meat, at the end of storage (11 days), CPW-treated beef exhibited the highest L* value (43.98), and maintaining a good texture. LTP treatment can also significantly reduce the content of polycyclic aromatic hydrocarbons (PAHs) in roast beef patties, especially PAHs containing 1–2 aromatic rings, which have a strong degradation effect by studying different LTP treatment methods, such as in-package cold plasma (ICP) pretreatment and cold plasma degradation (CPD) treatment. Compared with traditional heating methods, LTP treatment not only reduced the formation of more carcinogenic polycyclic aromatic hydrocarbons, but also inhibited the formation of PAHs by changing the composition of fatty acids [46]. In a direct DBD thawing application, Atani et al. [47] studied the effect of DBD on beef thawing by comparing DBD plasma, microwave thawing and conventional air thawing methods, and the results showed that DBD plasma can significantly shorten the thawing time (up to about 82% saving) and show obvious advantages in energy efficiency, reducing energy consumption by 95% compared with microwave thawing, which not only effectively inhibited microbial growth, but also maintained the structural integrity of meat. and there is no obvious protein denaturation or texture degradation. In addition to pork and beef, some scholars have also studied the application of LTP in the field of mutton preservation.

In addition to pork and beef, LTP has also been studied for mutton preservation. In an extract-modification approach, Khalili et al. [44] studied the antiseptic activity of free and nano-coated PPE obtained by cold plasma and ultrasound-assisted method in extending the shelf life of thigh lamb filling, and the results showed that PPE extract obtained by cold plasma-ultrasound-assisted extraction (CP-UAE) had significant antioxidant and antimicrobial activities. Wang et al. [42] also found that the combined treatment of cold plasma with TP could significantly inhibit the growth of the total number of viable bacteria, cryophilus, *Enterobacteriaceae*, and *Pseudomonas* in mutton.

### 3.2. Birds

For chicken, in a direct DBD treatment of chicken breast with oxygen and argon at low voltage (20 kV), Abdel-Naeem et al. [32] significantly reduced the number of bacteria (especially in the argon treatment group by more than 2 log10 CFU/g), and there was no significant change in thiobarbituric acid (TBA) and TVB-N (*p* > 0.05). The argon-treated samples performed best in sensory scores, especially in terms of color stability, and outperformed the oxygen-treated samples. Zhao et al. [33] used air plasma to treat chicken breast to extend its shelf life to 8 days, and the nitrogen plasma treatment group was 7 days, both significantly longer than the control group (3 days), indicating that both air plasma (air-CAP) and nitrogen plasma (nitrogen-CAP) could effectively inactivate microorganisms and extend the shelf life of chicken breast, but there were differences in the effects of the two: air plasma had stronger bactericidal ability (microbial logarithm reduction of 3.3 log CFU/g). In a direct treatment of chicken skin and breast fillet, Rossow et al. [48] reported that argon plasma yielded a peak average log reduction of 2.55 log CFU/cm^2^ across both test substrates, whereas air plasma only attained a value of 1.42 log CFU/cm^2^. In an in-package plasma treatment, Si Hyeon Roh et al. [49] showed that LTP treatment significantly inhibited the growth of *Salmonella* and further improved the inactivation effect through surface coatings such as whey protein. When combined with stirring, the inactivation rate of *Salmonella* in the chicken breast samples mixed with lettuce was significantly increased (2.8 log CFU/cm^2^).

For duck meat, in a combined procedure of LTP with sodium bicarbonate (NaHCO_3_), Jiang et al. [50] investigated the conformation, emulsification and rheological properties of duck myofibrillar proteins (MPs). The results showed that 0.6% NaHCO_3_ combined with LTP (50 V, 3 min) significantly increased the solubility (from 29.85% to 60.73%) and surface hydrophobicity, and reduced the turbidity and average particle size (from 1228 nm to 839.5 nm), indicating that NaHCO_3_ effectively reduced LTP-induced damage to duck protein structure and enhanced its functional properties.

### 3.3. Combined Procedures of LTP with Other Technologies

While standalone LTP treatment is capable of efficient microbial decontamination and quality retention, its preservative capacity is frequently constrained by the fleeting lifespan of reactive species, limited penetration depth, plus potential oxidative degradation triggered by over-intense processing. To address such drawbacks, LTP is more frequently integrated with antioxidants, ultrasound, salts, biopolymers and active packaging materials. Outcomes from these hybrid treatments stem from either synergistic or antagonistic interplay, so they should not be ascribed to LTP action in isolation. In a combined procedure of DBD plasma with 0.2% TP spraying, Wang et al. [42] reported that *E. coli* and total viable counts in mutton were significantly reduced, while elevations in TVB-N and pH were suppressed, resulting in a refrigerated shelf life extension of greater than 12 days. Analogously, prior to the marination of minced lamb, chlorogenic acid solution subjected to CP pretreatment yielded comparable effects [43], DPPH radical scavenging capacity increased 2.6-fold, total antioxidant capacity increased by 2.31-fold, carbonyl formation was significantly reduced (to 1.35 nmol/mg), heterocyclic amines and poly cyclic aromatic hydrocarbons were reduced by more than 50%. In a PAW–ultrasound synergistic treatment of chicken breast and skin [36], *E. coli* and *S. aureus* reductions reached 1.33 and 0.83 log CFU/mL, significantly higher than only treated via PAW (0.46 and 0.33 log CFU/mL), which was attributed to ultrasound-induced pore formation promoting ROS infiltration without changing protein, lipid, or texture properties.

When cold plasma was combined with sodium bicarbonate, the gel strength of duck myofibrillar protein was enhanced by 2.61-fold, the total sulfhydryl content attained 40.57%, whereas carbonyl and dityrosine contents declined by 62.58% and 46.95%, respectively. These findings suggest that NaHCO_3_ alleviates LTP-mediated protein oxidation [37]. In pork sausages packaged with argon CP treated PBS/TPS film coated with 80% Makwaen essential oil [31], the shelf life at 25 °C was extended significantly and TBARS was reduced by 0.45 mg MDA/kg; similarly, in a combined procedure of PALA with DBD plasma on chicken breast [35], the shear force was reduced by 19.4% and the myofibril fragmentation index was significantly increased.

## 4. The Mechanism of Low-Temperature Plasma to Achieve Meat Preservation

### 4.1. Microbial Inactivation Mechanism

**Figure 1:**
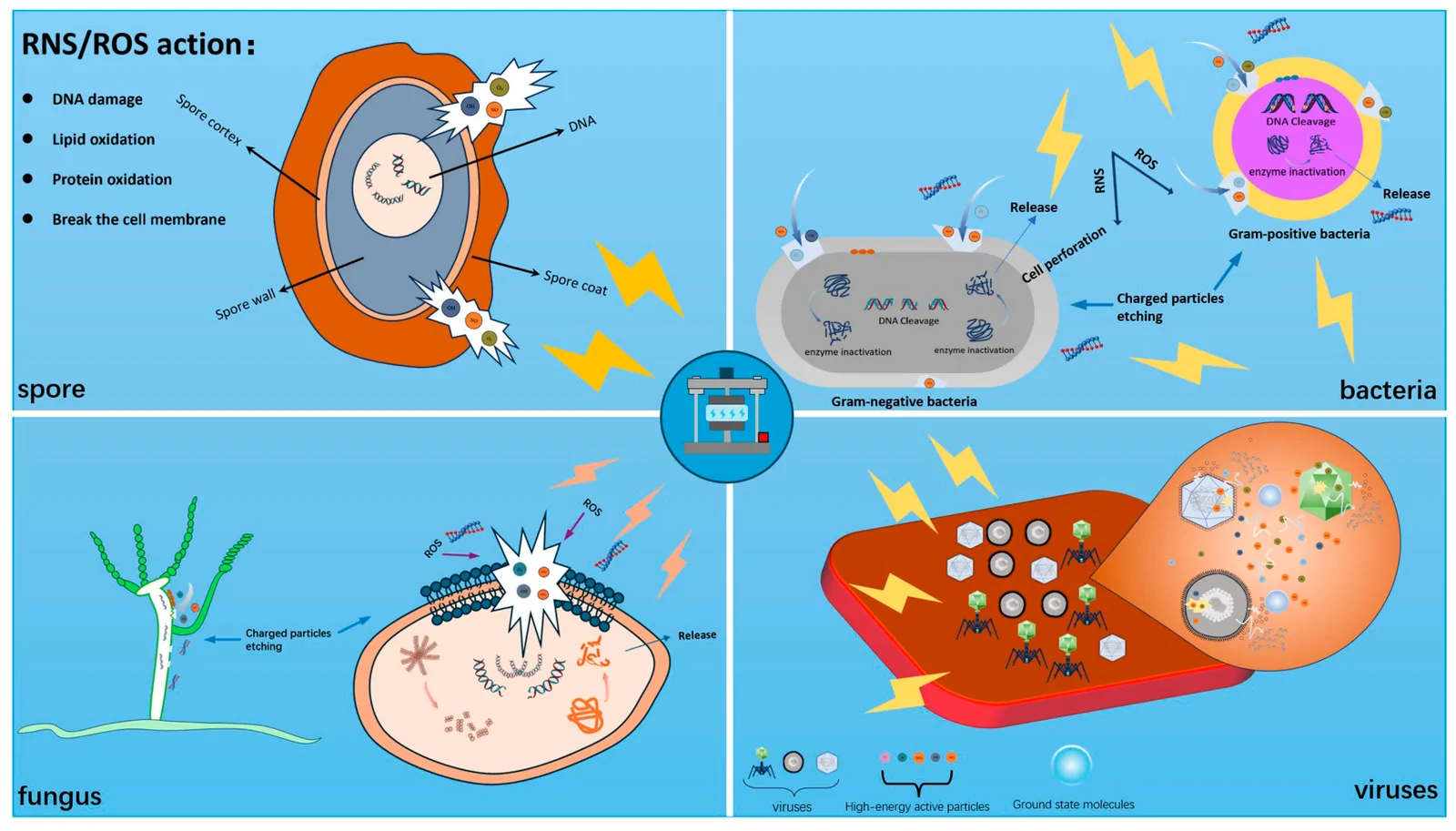
The mechanism of low-temperature plasma sterilization.

The inactivation mechanism of LTP on meat microorganisms is a multi-pathway complex process, which mainly uses the generated high-energy ROS and RNS, as well as ionization and electric field effects to inhibit or kill microorganisms (Figure 1). The following article elaborates in detail the mechanism of LTP on livestock and poultry meat preservation.

LTP produces a large amount of ROS and RNS during the discharge process, which have strong oxidizing ability and can damage the cell membrane structure of microorganisms, resulting in increased membrane permeability, which in turn causes the cell contents of microorganisms to leak or malfunction [51]. ROS mainly include hydroxyl radicals (·OH), superoxide anions (O_2_^−^), ozone (O_3_), and hydrogen peroxide (H_2_O_2_), while RNS includes nitric oxide (NO), nitrogen dioxide (NO_2_), nitrite (NO_2_^−^), and peroxynitrite (ONOO^−^). First, at the cell membrane level, ·OH and O_3_ in ROS are the main destructive factors. ·OH extracts hydrogen atoms from unsaturated fatty acids in cell membranes, triggering a lipid peroxide chain reaction to generate lipid peroxides, resulting in loss of membrane structural integrity, dramatic increase in permeability, and leakage of key substances such as intracellular electrolytes and nucleic acids; O_3_ directly oxidizes the phospholipid bilayer and destroys the membrane barrier function. Gram-negative bacteria are more sensitive to this attack due to their thin cell membranes and lack of thick peptidoglycan protection, and the high concentration of OH and O_3_ produced by air plasma reduced the logarithm of Gram-negative bacteria such as *Pseudomonas* by 3.3 log CFU/g in chicken breast LTP treatment [52]. The NO in RNS can bind to membrane proteins, change the membrane potential and ion transport system, and synergize with ROS to aggravate membrane damage, and the nitrification reaction between NO and *Campylobacter jejuni* membrane protein increased its inactivation efficiency by 1.5 times compared with air plasma when chicken skin was treated with argon plasma [8,48].

In the protein damage pathway, ROS and RNS modify amino acid residues through oxidation and nitrification, leading to loss of enzyme activity or structural protein denaturation. ·OH can oxidize the sulfur group of cysteine to form disulfide bonds, or oxidize methionine to form sulfoxide, destroying the enzyme activity center; ONOO^−^ changes the three-dimensional conformation of proteins by nitrifying tyrosine residues. LTP treatment will inactivate key metabolic enzymes such as succinate dehydrogenase in microorganisms due to the above modifications, block the tricarboxylic acid cycle and ATP production, and ultimately inhibit their growth and reproduction [53]. For fungi, RNS can also degrade chitin and β-glucan in the cell wall, weaken the mechanical strength, and effectively inhibit spore germination with ROS attack on the cell membrane [54].

Nucleic acids, as genetic material targets, are synergistically damaged by ROS and RNS. ·OH attacks DNA bases to produce oxidation products such as 8-hydroxydeoxyguanosine, and O_3_ causes phosphodiester bond breakage to form single/double-strand gaps. ONOO^−^ triggers DNA deamination and glycophosphate backbone damage, interfering with base pairing and replication transcription. The RNA genome of the virus is more susceptible to damage by ·OH and ONOO^−^ due to the lack of effective protection from the protein capsid. After LTP treatment of foodborne viruses, capsid proteins lose their ability to adsorb host cells due to oxidative denaturation, and RNA strand breaks lead to infectivity loss [55].

The differences in inactivation efficiency of different microorganisms stem from the structural and metabolic characteristics: although the thick peptidoglycan layer of gram-positive bacteria can delay ROS penetration, RNS can enter the cell through the peptidoglycan gap and act on the plasma membrane together with ROS [56]. The cell wall components of fungal mycelium and spores (e.g., chitin and dextran) became specific targets of RNS, and their degradation led to a decrease in the mechanical strength of the cell wall [57]. The gas composition and treatment parameters significantly affected the distribution of active species and the inactivation effect: the air plasma was rich in O_2_, which was conducive to the formation of O_3_ and ·OH, and had outstanding effects on gram-negative bacteria and fungi. Nitrogen plasma focuses on NO and ONOO^−^ production, which was more advantageous in virus and spore inactivation [58]. In summary, ROS and RNS form a complementary oxidation-nitrification network in cold plasma, and achieve broad-spectrum inactivation of bacteria, fungi, viruses and even spores through the synergistic effects of membrane lipid peroxidation, protein function inactivation and nucleic acid damage.

### 4.2. Effects on the Physicochemical Properties of Meat

#### 4.2.1. pH Value

Freshly slaughtered meat is slightly alkaline and has a pH between 7.0–7.2. At this time, muscle cells are still alive, but due to the loss of blood supply to the muscles, they gradually shift from aerobic metabolism to anaerobic metabolism, and continue to produce lactic acid, which accumulates in the muscles, causing the pH value to continue to drop from close to physiological levels (about 7.0). In this natural process, if the meat is treated with LTP, some new changes are introduced.

In untreated fresh meat samples, the pH drop is mainly affected by the rate and amount of lactate production. In the fresh meat samples treated with LTP, the surface layer of the meat reacts with the meat tissue components due to exposure to active species such as ROS and RNS, resulting in a slight decrease in pH [59]. In the subsequent storage stage, plasma treatment can effectively delay the spoilage process by inhibiting microbial growth and slowing down the pH increase caused by microbial metabolism during storage [60]. In summary, although LTP treatment has a certain effect on the local pH of fresh meat, it will not destroy the gradual decrease of pH during its natural change process, and can increase the stability of pH, which is conducive to meat quality control and shelf-life extension.

#### 4.2.2. Water Retention

Water is one of the most important components of fresh meat, generally accounting for 70–75% of the meat weight. LTP treatment can have a significant impact on the moisture state of meat, and studies have shown that LTP treatment can cause rapid dehydration of the surface of ham, resulting in a decrease in its moisture content [61]. Fresh untreated meat usually has a high water-holding capacity because the muscle tissue is structured intact and the myofibrils and sarcoplasmic network system can effectively fix water. The water distribution of meat treated with LTP showed significant changes: the proportion of free water increased, which was manifested by an increase in juice loss rate, especially during thawing or heating, when the water retention capacity was significantly reduced due to cell structure damage. The effects of LTP treatment on meat are mainly reflected in the change of the ratio of free water to bound water, the decrease of water-holding capacity and the microscopic damage of tissue structure [62]. In addition, the active species produced by the action of LTP may also trigger the oxidation or cross-linking of proteins, change the conformation and hydrophilicity of proteins in muscle, thereby affecting the ability of proteins to bind to water, such as plasma-activated water can induce oxidation and conformational recombination of PSE-like chicken proteins without directly touching the surface of food, and significantly change its hydrophobicity, aggregation state and microstructure, thereby affecting the binding and retention ability of water [63].

#### 4.2.3. Lipids

**Figure 2:**
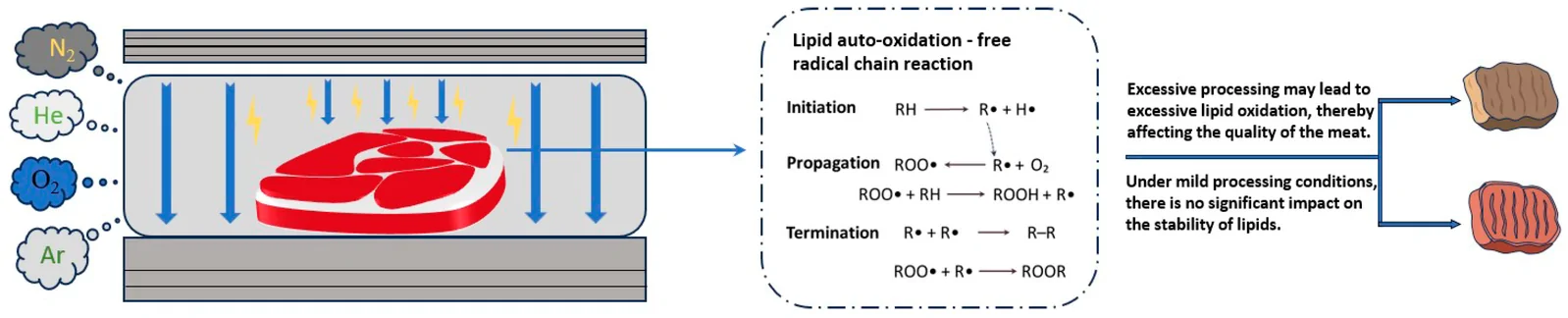
The mechanism of lipid oxidation caused by low-temperature plasma.

Lipids are a class of organic compounds mainly composed of carbon, hydrogen, and oxygen, which are insoluble in water and soluble in organic solvents [64]. In the untreated state, lipids in fresh meat are mainly in the form of triglycerides, phospholipids and a small amount of free fatty acids, which play an important role in muscle cell membrane structure maintenance, flavor production and energy storage. However, these lipids, especially phospholipids rich in polyunsaturated fatty acids (PUFAs), are more sensitive to oxidation. LTP triggers the radical chain oxidation reaction of unsaturated fatty acids (especially polyunsaturated fatty acids) in lipid molecules by producing ROS and RNS such as hydroxyl radicals and nitric oxide (Figure 2). Radical chain oxidation generally divided into three stages: initiation, propagation and termination [65]. This process is first manifested by the formation of hydroperoxides, a primary oxidation product, resulting in an increase in peroxide value (PV), followed by further decomposition into secondary products such as aldehydes (e.g., hexanal, nonanal), ketones, carboxylic acids, and volatile compounds. Wang et al. detected the oxidation products of lipids in mutton and verified the dominant role of lipid oxidation in the formation of mutton flavor at different ages [66]. Pérez-Andrés et al. [67] also used the TBARS value as an indicator of the degree of lipid oxidation to evaluate the effect of plasma on lipid oxidation during storage in commercially packaged mackerel fillets.

TBARS value was used to systematically measure and analyze the lipid oxidation in beef patties by using LTP at different voltages (50, 60, 70 kV) to investigate the oxidation of lipids [68]. Gao et al. [59] further analyzed the role of rosemary extract (natural antioxidant) in inhibiting the oxidation reaction based on the determination of TBARS value to evaluate the effect of LTP treatment on lipid oxidation, and experimentally used dielectric resistance discharge plasma technology to encapsulate the minced chicken patties for 70 kV and 180 s, and set up a control group (no CP, no rosemary) and the treatment group (CP, with/without rosemary) to compare, and monitored the TBARS value of the samples at 0 days and 5 days of storage. The results showed that LTP significantly promoted lipid oxidation at 70 kV, and rosemary extract could effectively inhibit lipid oxidation. In addition to the TBARS value, there are also some equivalent indicators that can also reflect the degree of oxidation of lipids. For example, in the experiments of Sarangapani et al., a variety of advanced analytical methods such as Fourier transform infrared spectroscopy (FTIR), fatty acid composition analysis (GC-FID), nuclear magnetic resonance hydrogen spectroscopy (^1^H NMR), and peroxide value (PV) were used to systematically evaluate the lipid oxidation process of LTP treatment, and the oxidation reaction caused by LTP treatment of beef and dairy lipids was systematically demonstrated. The results confirmed the central position of the Criegee mechanism in lipid oxidation and found that beef lipids are more sensitive to oxidation than dairy lipids (higher proportion of unsaturated fatty acids) [69].

Kim et al. [70] used pure helium and 0.3% oxygen added to helium to treat pork tenderloin using DBD plasma. The results showed that the TBARS value of the plasma treatment group with oxygen was significantly higher than that of the pure helium group and the control group, indicating that the introduction of oxygen significantly exacerbated lipid oxidation. Li et al. [71] discussed the freshness preservation effect of LTP and argon modified atmosphere packaging on meatball products. The results showed that the oxidation effect of LTP treatment was related to the treatment time and voltage, and the longer the treatment time, the higher the discharge intensity, the more active species produced, and the greater the oxidation risk. And argon as a gas medium also makes the treatment milder and more controllable.

#### 4.2.4. Protein

The process of LTP acting on proteins is a continuous chain of reactions triggered by chemical modifications, which in turn lead to functional regulation. LTP can trigger oxidation, fracture and cross-linking reactions of protein molecules through the interaction between active species and proteins, change the spatial conformation and surface physicochemical properties, and then affect the functional properties of proteins such as solubility, emulsification, foaming, and gelability [72]. LTP can react with protein molecules in a variety of non-thermal conditions, resulting in multi-level changes in the chemical structure and conformation of protein molecules. The active species such as ROS and RNS can react with protein molecules at room temperature, inducing chemical modifications such as oxidation, degradation, and cross-linking [73].

**Figure 3:**
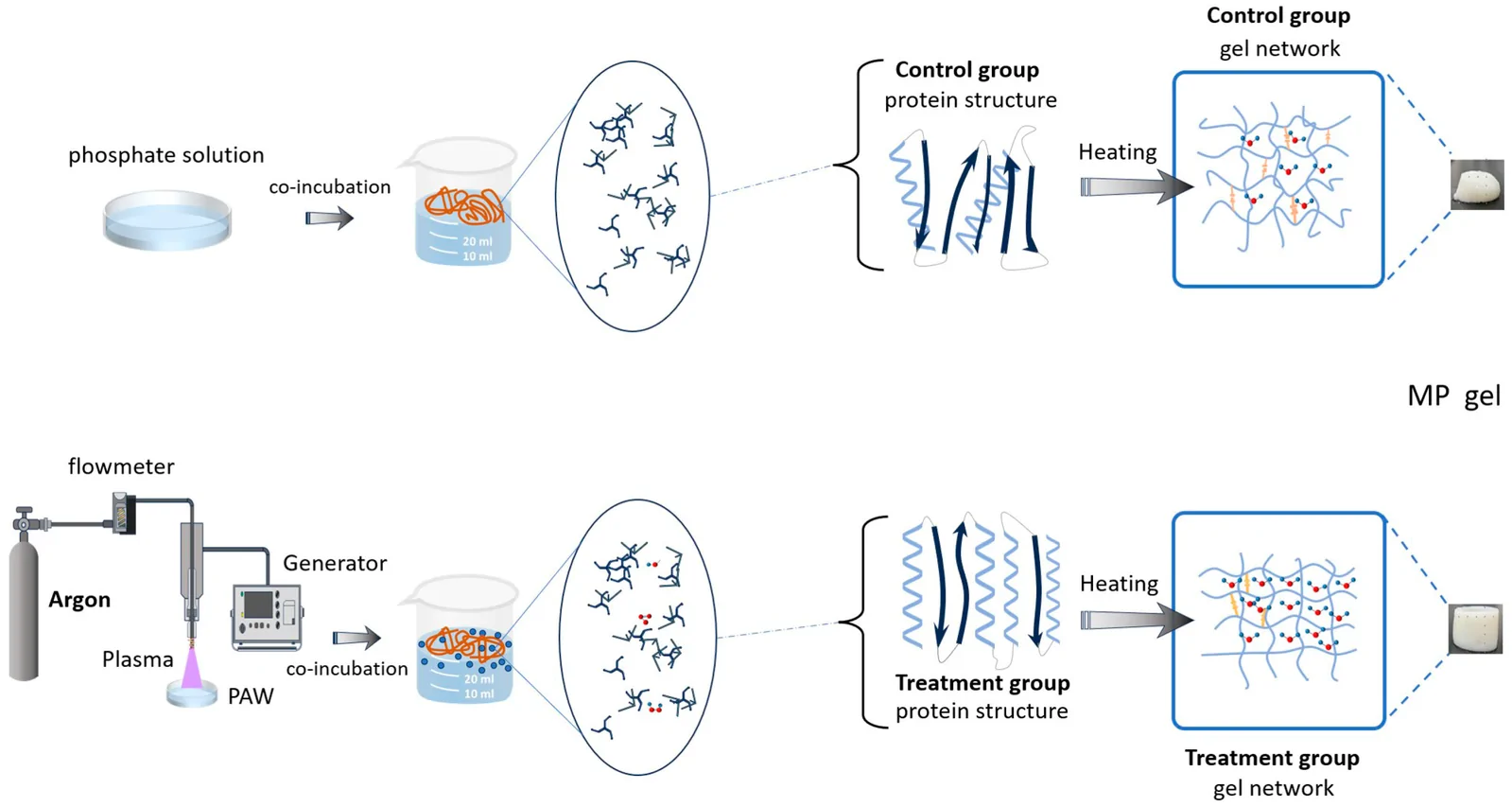
The mechanism of protein oxidation caused by low-temperature plasma.

During the treatment of LTP, a variety of reactive species can be produced to interact with protein molecules, and these active species will attack the amino acid residues in the protein, especially aromatic and sulfur-containing amino acids, causing chemical modifications such as side chain breaks, skeleton breaks, cross-linking, and polymerization, resulting in changes in protein molecular structure, inducing changes in the secondary structure of proteins, such as the transformation of α-helix to β-fold and random coil, resulting in the expansion of protein molecular structure. Expose more hydrophobic and functional groups, thereby enhancing functional properties such as emulsification, foaming, gelling, etc [74]. J. Li et al. [75] systematically investigated the effects of argon low- PAW on the structure and function of chicken MP, and found that the main active species in PAW changed the secondary and tertiary structure of proteins through oxidative modification interaction with hydrogen bonding and electrostatic interaction, promoting the increase of α-helix content and the transformation of β-sheet to β-corner, thereby enhancing protein order and gel strength. At the treatment was 16 min, the gel strength was the highest (0.53 N), the cooking loss was 30.38%, the proportion of bound water increased for 0.26%, which indicated that PAW can alter protein structure and enhance MP’s functional properties (as shown in Figure 3).

### 4.3. Effect of Low-Temperature Plasma on the Organoleptic Properties of Meat

#### 4.3.1. Chromaticity

Before and after LTP treatment, the color of meat will change significantly and regularly, which is reflected in the adjustment of three key indicators, namely L* value, a* value and *b* value, and the direction and degree of change are affected by the treatment parameters (such as power, time, gas type) and the characteristics of the meat itself. The L* value of the treated beef patties decreased significantly at 60 and 70 kV, and decreased with the increase of voltage, but remained relatively stable during storage. There was no significant difference between the treatment group and the control group. The a* value decreased significantly at all voltages, and continued to decrease with the extension of storage time, and the decrease was most obvious at 70 kV [68]. Cheng et al. [76] conducted a study on red meat, and showed that plasma activation solution had a significant effect on the chromaticity of metMb and oxymyoglobin (oxyMb), and the active species generated in the activation solution could induce myoglobin oxidation, and the short-term treatment (≤2 min) had no significant effect on the color of metMb, while after more than 4 min treatment, the reddish-brown color gradually faded and increased to 8–10 min appears slightly green, while for oxyMb, the color changes from bright red to reddish-brown at ≤4 min, indicating that it is oxidized to metMb, and further binds to H_2_O_2_ at 8–10 min, resulting in heme (heme) degradation and green. The effect of indirect plasma treatment on the color of fresh pork was that the L* value increased slightly after treatment, but the difference was not significant. The a* value decreased significantly, and the decrease was greater in the longer treatment time, but the a* value gradually rose in the subsequent storage process, while the a* value of the control group gradually decreased, and the two tended to be close in the later stage of storage. The b* value increased significantly after long-term treatment, which was presumed to be related to the oxidation of myoglobin triggered by free radicals in the plasma to produce methemoglobin [60].

#### 4.3.2. Flavor and Taste

The process of flavor formation meat is very complicated, but ultimately, it is the result of the interaction between the generation of volatile compounds and sensory characteristics, and it is also inseparable from the influence of factors such as intrinsic components and external processing. During the LTP processing, the flavor of meat mainly comes from the lipid and the oxidative decomposition of proteins [77]. The fat in the meat is one of the most important flavor precursors, and it will produce volatile flavor substances such as aldedes, ketones, and alcohols during the oxidation process, which will have a positive or negative impact on the flavor of the meat, and inert gases such as argon less negative impact on the flavor than oxygen [9]. During the LTP action process, the oxidation of proteins causes the accumulation of carbon groups, and may interact with the by-products produced by lipid oxidation, thus affecting the texture and flavor of the meat [78]. Apart from the role of important components in meat, different kinds of meat also have a certain impact on flavor because of the differences in fatty acid composition and protein [79]. Chicken is mainly composed of a large amount of fat oxidation products (such as aldehydes and ketones) and protein decomposition products (as leucine), with spicy and fishy aromas as the main flavors; beef contains more esters and alcohols, leaning more towards grassy and fruity flavors;, on the other hand, is dominated by degradation products of branched-chain amino acids (such as valine, leucine), aldehydes, with a distinct fatty and sweet flavor; duck meat, however, is characterized by its higher content of nitrogen and sulfur volatiles, in addition to its strong spicy flavor it also has a weaker hydrocarbon odor [80].

As a new non-thermal processing technology, LTP is widely used in the field of meat preservation and quality optimization, which significantly changed the flavor composition of meat by regulating the oxidative decomposition of lipids and proteins. Under LTP treatment, the core mechanism of meat flavor is still the oxidative decomposition of lipids and proteins, but the participation of active particles makes this process more controllable, which in turn leads to specific changes in the types and contents of flavor-bearing substances. At the lipid oxidation level, unsaturated fatty acids in meat are key flavor precursors, and the ROS produced by LTP can moderately initiate oxidation reactions to generate characteristic volatile substances such as aldehydes, ketones, and alcohols. At the same time, if inert gases such as argon are used as discharge agents, excessive oxidation can be effectively inhibited and the accumulation of hexanal and other bad odor substances can be reduced. Research has shown that PAW effectively preserved the flavor profile by inhibiting the formation of key off-odor compounds (e.g., 1-nonanol, butanal) [81]. The significant differences in metabolites were likely resulted from the presence of nitrite generated via LTP, as nitrite contributed to preservation and delayed the formation of undesirable odors.

The regulation of protein oxidative decomposition is another key path for LTP to improve flavor, and this regulatory effect is also directly reflected in the sensory evaluation results of different meat varieties. LTP treatment will lead to changes in protein structure and promote the accumulation of carbon groups, and its oxidation products can interact with lipid oxidation by-products (such as malondialdehyde MDA) to regulate the degradation of amino acids. In beef, LTP treatment with appropriate strength can promote the degradation of branched-chain amino acids, increase the content of fruity substances such as 3-methylbutyraldehyde and 2-methylbutyraldehyde, and enhance the unique flavor of beef with grassy aroma and fruity aroma. Sensory evaluation further revealed that moderate LTP treatment markedly enhanced the grassy and fruity aroma notes of beef, raised overall flavor acceptability, and did not generate any unpleasant oxidative off-flavor [82]. Over-processing will lead to excessive oxidation of proteins and the formation of odorous substances such as sulfides [79], this aligns with sensory findings that excessive LTP exposure gives rise to a pronounced pungent odor and lowers taste acceptability in beef products [83]. For duck meat, LTP treatment can inhibit the formation of undesirable volatiles containing sulfur and nitrogen, significantly reduce the intensity of strong spicy taste, reduce the odor caused by hydrocarbons, and optimize the overall flavor coordination. Sensory panel tests further confirmed that optimized LTP treatment effectively mitigated the characteristic fishy and pungent off-flavors of duck meat, balanced its flavor intensity, and yielded higher overall sensory scores than the untreated groups [81].

Due to the differences in fatty acid composition and protein type, the flavor changes of different meats after LTP treatment showed significant species-specificity, which is also manifested in distinct sensory performance. After LTP treatment, the proportion of aldehydes and ketones in the fat oxidation products was more balanced, and the content of amino acid decomposition products such as leucine was stable, maintaining its unique fishy aroma and spicy flavor base [84]. In addition, LTP treatment can also enhance the binding ability of myofibrillar proteins to flavor compounds, improve flavor stability, and maintain good flavor quality during storage [85]. Long-term sensory monitoring during storage showed that LTP-treated meat samples maintained more stable flavor and accumulated less off-flavor than control samples, confirming the practical flavor-improving effect of LTP technology from a sensory perspective.

In summary, LTP realizes the directional optimization of meat flavor by precisely regulating the oxidative decomposition of lipids and proteins, and its core is to retain positive odor substances and inhibit the formation of bad odors by controlling the treatment parameters and discharge agent types. This technology provides a new path for the precise regulation of meat flavor quality, and in the future, it is necessary to further optimize the processing parameters to achieve personalized improvement of different meat flavors and match the optimal sensory quality requirements of various meat products.

#### 4.3.3. Texture

As one of the core evaluation indicators of meat quality, texture directly determines consumers’ chewing experience and acceptance, which are mainly regulated by myofibrillar protein structure, intermuscular fat content and cell integrity. LTP changes protein conformation and cell state at the molecular level through the interaction between active particles and meat components, which in turn has a significant regulatory effect on meat texture, and this effect is significantly different due to treatment parameters and meat varieties. Apart from instrumental texture detection, the core regulatory mechanism of LTP on meat texture focuses on the structural modification of myofibrillar proteins. ROS, reactive nitrogen and other particles produced by LTP can attack amino acid residues in protein molecules, trigger disulfide bond breaking and re-crosslinking, hydrogen bond remodeling and other reactions, and change the solubility and aggregation state of proteins. LTP treatment with appropriate intensity can moderately depolymerize myofibrillar proteins, reduce the density of muscle fiber bundles, and significantly improve meat tenderness [38]. For instrumental assessment of LTP-induced tenderness changes, Warner–Bratzler shear force (WBSF) measurement, texture profile analysis (TPA)/double compression, and penetration tests have been the primary approaches. In chicken breast treated with PALA-DBD, WBSF measurement showed a 19.4% reduction in shear force relative to the deionized water-treated group, accompanied by a significant increase in the myofibril fragmentation index [35]. In beef myofibrillar protein gels, moderate LTP exposure (60–90 s) resulted in gel strength increasing from 2200 g·mm to 5800 g·mm (more than doubling, TPA/double compression), with significant improvements in textural attributes such as springiness and cohesiveness (approximately 15–20% increase) [86]. These findings indicate that the extent of LTP-induced tenderness improvement depends strongly on treatment parameters, meat type, and the instrumental testing method used. In the pork LTP treatment experiment, after 70 kV DBD-CP treatment, the shear force of pork decreased from 35.23 N to 33.09 N (approximately 6.1% reduction, WBSF measurement), the myofibrillar fragmentation index (MFI) increased from 41.86 to 44.17, the surface hydrophobicity of myofibrillar protein was significantly improved, and emulsifying activity index (EAI) and emulsifying stability index (ESI) increased by 66.67% and 54.21%, respectively, and the texture was significantly improved [87]. In this study, tenderness was assessed via shear force measurement and the MFI. The slight drop in shear force together with the rise in MFI points to a tenderizing effect linked to myofibrillar depolymerization and structural disruption. Concurrent increases in EAI and ESI further imply that LTP altered the interfacial and hydration properties of myofibrillar proteins.

In addition to protein modification, the regulation of meat cell structure by LTP also indirectly affects the texture characteristics. The high-energy particles generated by LTP can destroy the integrity of the cell membrane on the surface and superficial layer of meat, allow the moderate exudation of water and small molecules in the cell, and promote the interaction between sarcoplasmic protein and myofibrillar protein, forming a more stable protein gel network. This gel network effectively encapsulates water and fat, further enhancing the water retention and elasticity of meat. However, it should be noted that excessive LTP treatment can lead to excessive cell membrane damage, causing excessive oxidation and aggregation of proteins, which will aggravate juice loss and increase the hardness and elasticity of meat [88]. This dual effect was quantified by TPA/double compression testing. In pork exposed to LTP for 6 min, hardness, springiness, and chewiness did not differ significantly from the control. However, extending treatment to 9 min raised hardness, springiness, and chewiness by roughly 35%, 17%, and 42%, respectively. Such a rebound indicates that LTP-induced tenderization is dose-dependent and has an upper threshold, beyond which texture deterioration occurs [38]. In addition, the type of discharge unit and the processing environment of LTP will also affect the texture regulation effect. When using argon, nitrogen and other inert gases for LTP treatment, it can reduce the over-generation of ROS, avoid excessive oxidation of proteins, and achieve precise texture optimization compared with air discharge. Treatment in a low temperature and low humidity environment can reduce the rapid evaporation of water on the surface of meat, further improving the optimization effect of water retention. At the same time, the synergistic effect of LTP treatment and subsequent processing technology cannot be ignored, such as LTP pretreatment combined with low-temperature storage, which can effectively delay the texture deterioration of meat and extend the shelf life.

## 5. Conclusions

This review summarizes recent advances in LTP for livestock and poultry meat preservation, and elucidates its mechanisms in microbial inactivation, oxidation regulation, and meat quality maintenance. As a new non-thermal processing technology, LTP has shown significant advantages and application potential in the field of meat preservation. Multiple forms of discharge produce active substances such as ROS and RNS, which achieve efficient sterilization and significantly reduce the microbial load of meat with the help of multi-path action that destroys microbial cell membranes, damages proteins and nucleic acids. At the same time, LTP can regulate the oxidation process of lipids, delay protein denaturation, maintain the pH stability, color and texture of meat, effectively extend the shelf life of meat, and has the characteristics of no chemical residues, low energy consumption, and environmental friendliness, making up for the shortcomings of traditional preservation technology. In practical applications, the LTP treatment effect is significantly affected by factors such as discharge mode, gas medium, and process parameters. At present, the research on the mechanism of LTP in livestock and poultry meat preservation still needs to be deepened, and the problems of optimization of adaptability of different meat breeds and process standardization in industrial applications still need to be solved. In the future, it is necessary to further focus on the precise analysis of the mechanism of LTP, optimize the discharge parameters and gas medium combination, and the personalized preservation scheme of different livestock and poultry products, so as to provide more efficient technical support for reducing meat circulation loss and ensuring food safety.

## Figures and Tables

**Table 1:** Types of low-temperature plasma with different forms of discharge.

Types	Principle	Advantages	Disadvantages	Application	References
Dielectric barrier discharge (DBD)	By inserting an insulating dielectric between the electrodes and applying high-frequency and high-voltage alternating current, a large number of micro-discharge wires are formed after breaking down the gas, producing a low-temperature plasma containing electrons, ions, free radicals, etc.	a. High-density non-thermal plasma can be produced at room temperature and pressure, with simple process and low energy consumption. b. The equipment structure is simple. c. The discharge characteristics and reaction process can be flexibly adjusted.	a. Uneven discharge, resulting in uneven distribution of treatment space. b. The energy utilization efficiency is limited. c. Limited penetration depth.	a. Microbial inactivation and surface sterilization. b. Toxin degradation. c. Enzyme inactivation and browning control. d. Food drying and structural modification.	[13,14]
Corona discharge (CD)	In extremely inhomogeneous electric fields, the surface of electrodes (e.g., tips, thin wires) with a small radius of curvature exceeds the gas ionization threshold due to the local electric field strength, which ionizes the air molecules.	a. It can achieve partial discharge at lower energy and consume less energy. b. The device has a simple structure, fast start-up and flexible control.	a. Not suitable for large-scale ionization applications b. Ozone and nitrogen oxides produced during the discharge process can cause environmental pollution.	a. Food drying and thawing are accelerated. b. Surface sterilization and preservation. c. Improve food structure and functional properties.	[15,16]
Microwave discharge (MD)	The gas is excited by high-frequency electromagnetic waves from 300 MHz to 300 GHz (commonly 2.45 GHz), causing free electrons to accelerate and collide with gas molecules in an alternating electric field, initiating ionization when the energy exceeds the ionizing energy.	a. High excitation efficiency. b. Suitable for working with heat-sensitive materials. c. It avoids electrode corrosion and contamination problems in traditional discharge methods. d. The microwave plasma is evenly distributed in space.	a. The cost of equipment is relatively high. b. The plasma region is limited by the microwave field distribution. c. Sensitive to gas purity and flow rate.	a. Structural modification of food ingredients. b. Efficient treatment of food waste.	[17,18]
Frequency discharge (FD)	Alternating current is generated in the electrode or induction coil through alternating current in the RF band (3 kHz–300 MHz), which accelerates the free electrons in the gas by excitation, collides with the gas molecules and ionizes them.	a. High-density plasma can also be generated in low-pressure environments. b. Low requirements for electrode materials. c. High ionization efficiency and good compatibility with material processes.	a. Rely on inert gases (e.g., Ar, He) to maintain stable discharge. b. It needs to be equipped with high-frequency RF power supply, vacuum system and precision control device, and the equipment cost is high.	a. Disinfection treatment of packaging materials and food surfaces. b. Food drying and moisture control. c. Food structure and function modification.	[19,20]

**Table 2:** Application of low-temperature plasma technology in the field of livestock and poultry preservation.

Type	Treatment Configuration	Treatment	LTP	Parameters	Main Findings and Limitations	References
Chicken	Direct	Gases: O_2_, Ar Surface treatment.	DBD	Voltage: 20 kV; Processing time: 3 and 5 min	The total number of colonies, cryophilus, *Staphylococcus aureus*, and *Enterobacteriaceae* decreased significantly (Ar treatment decreased by >2 log CFU/g). There was no significant difference between TBA and TVB-N (*p* > 0.05). The brightness (L*) of the oxygen group increased from 48.12 to 52.80, and yellowness (b*) increased from 11.08 to 13.76, the redness (a*) decreased 1.48. The shear force decreased, and the tenderness of the argon group increased more obviously.	[32]
Chicken breast	Direct	Gas: Air and N_2_ Surface treatment, refrigerated storage (4 °C).	Cold atmospheric pressure plasma jet (CAPPJ)	Input power 800 W, processing time 0, 5, and 10 min, the distance between the plasma electrode and the sample stage was 15 cm.	It significantly delayed the rise of TVB-N and moderated the changes in pH, and extended the shelf life from 3 days to 8 days (Treated for 10 min under air as gas). the sensory attributes were basically maintained. At the end of the shelf life after treatment, the dominant flora was changed from spoilage bacteria to *Bacilloids*, which improved microbial diversity and safety.	[33]
Chicken breast and skin	Direct	Gas: N_2_ Surface treatment, stand for reaction and refrigerate.	Floating electrode-dielectric barrier discharge (FE-DBD)	Voltage 2.8 kV, frequency 47 kHz, processing time: 10–60 min, discharge gap 3 mm	A reduction of *Salmonella* Enteritidis by 0.45 and 0.17 log CFU/g in chicken breasts and skin, respectively. The pH value of chicken breast increased from 5.87 to 6.02 after 60 min, had little effect on color, and shear force. The TVB-N content increased, ranging from 5.15 to 5.31 mg/100 g in chicken breasts and 7.24 to 7.30 mg/100 g in chicken skin.	[34]
Chicken breast	Combined	Gas: Air Sequential processing: plasma-activated lactic acid solution (PALA), then placed in a packaging box.	DBD	PALA preparation time was 0–180 s, PALA treatment (0–10 min), the voltage was 0–160 kV, the treatment time was 0–5 min, discharge gap 30 mm.	Effectively inhibits the bacteria count for 1.24 lg CFU/g under the PALA preparation time was 90 s. On the fourth day, the pH value of the control group rose to 6.36, while the pH values of the plasma-treated were 5.91, 6.16, and 5.67, respectively, which were significantly lower (*p* < 0.05) than that of the control group.	[35]
Chicken breast and skin	Combined	Gas: Ar First, PAW was generated by Ar discharge, and then combined with ultrasound.	The single-electrode non-thermal atmospheric pressure underwater plasma jet.	Voltage: 6.8 kV, frequency: 1.5 kHz, air flow: 3 slm, discharge 6.5 min to generate PAW. Combined with 40 kHz, 220 W ultrasound, 40 °C for 60 min.	PAW—ultrasonic synergy can increase the *E. coli* decreased by 1.33 log CFU/ml and *S. aureus* decreased by 0.83 log CFU/ml, which was significantly higher than that of PAW alone (0.46 and 0.33 log CFU/ml). After treatment, the pores on the surface of the muscle increased, promoting ROS infiltration, did not change the protein, lipid or texture, the color difference was *ΔE* < 5, and the sensory quality remained good.	[36]
Duck myofibrillar protein (MP)	Combined	Gas: Air Cold plasma + sodium bicarbonate (SB) synergistic treatment. The treated MP was placed in a 15 mL beaker and heated at 80 °C for 30 min to induce gelation.	DBD	The processing time was 3 min (50 V), and the SB concentration was 0.2%, 0.4%, and 0.6%.	Compared to control group (DBD), the gel strength of CP-SB_0.6_ group increases 2.61-fold, total sulfhydryl content was 40.57%, reductions in carbonyl and dityrosine contents by 62.58% and 46.95%, respectively.	[37]
Pork	Direct	Gas: Air Surface treatment.	Piezoelectric direct discharge plasma (PDD)	Input voltage: 15 V, frequency 50 kHz, Treatment durations: 0, 6 and 9 min.	Overprocessing (9 min) led to a peroxide value of 1.88 meq/kg, protein carbonyls of 17.80 nmol/mg protein, and aldehydes of 13.10 pmol/mL homogenate, accelerating deterioration of the oxidative quality of pork.	[38]
Pork	Direct	Gas: Air Surface treatment, refrigerated storage (4 °C).	PDD	Voltage: 12 V, Frequency: 50 kHz, Various treatment durations (0, 1, 3, 6, and 9 min) were applied.	The 3 min CAP treatment led to a significant reduction of total viable counts levels (up to 1.4 log CFU/g reduction) on days 0, 4, and 6. CAP treatments longer than 6 min maintained significantly lower levels of total viable counts, *Pseudomonas* spp., and *Enterobacteriaceae* throughout storage, suggesting improved microbiological stability during 8 days of refrigerated storage.	[39]
Ice-fresh Pork	Packaging	Gases: air, N_2_ Pectin was modified by DBD and cross-linked with chitosan to prepare aerogel pads for refrigerated packaging of pork.	ACP	Power 400 W, gas flow rate 25 L/min, electrode distance 5 cm, processing time 60–90 s.	Compared to the control group, nitrogen plasma treated 90s (N90C) effectively reduced the formation rate of TVB-N by 63.40%. Significantly reduced the values of TBARS and prolongs the shelf life, has better moisturizing properties, the a* was 4.60 on the 10th storage days, which was higher than control group (2.63).	[30]
Beef *Musculus longissimus dorsi*	Direct	Gas: O_2_, Ar Surface treatment, refrigerated at 4 °C.	ACP	Voltage: 25 kV, Frequency: 42 kHz, Processing time 15 and 30 min.	ACP (15–30 min) reduced beef steak microbes by average 2.00 log CFU/g. The 30 min 50% O_2_-50% Ar group reached pH 5.05 and *A_w_* 0.845. Its TBARS was 2.58 mg MDA/kg, and microbial counts stayed lowest over 7-day storage to extend shelf-life.	[40]
Beef	Direct	Gas: O_2_ Indirect treatment, closed container, low-temperature gas circulation.	DBD	The voltage had a differential pattern with a peak of 15 kV, processing time 0–300 min, cold chain temperature approx. 4 °C	The total number of bacteria on the surface of beef was significantly reduced (3.6–4.1 log CFU/g) in both cold chain and room temperature. Cold chain sterilization has better effect and maintains stable meat quality, with no obvious color and sensory changes.	[41]
Mutton	Combined	Gas: Air Surface spraying treatment + cold plasma (CP) irradiation, refrigerated storage.	DBD	Voltage: 75 kV, 150 s treatment time, combined with 0.2% tea polyphenol (TP) spray.	DBD combined with TP significantly reduced the number the total viable count to 5.95 log CFU/g after 13 d, inhibited the increase of TVB-N and pH, delayed lipid oxidation, improved sensory scores.	[42]
Mutton	Combined	Gas: Air The minced mutton was marinated in CP-treated chlorogenic acid solution for 4 h and then shaped and baked.	DBD	Power: 40 W, air flow rate 30 L/min, processing time: 15 min, output voltage: 8 kV, frequency: 20 kHz.	The chlorogenic acid solution treated with CP significantly improved the brightness and tenderness of the patty, increased the scavenging capacity of DPPH free radicals by 2.6 times, and increased the total antioxidant capacity by 2.31 times. Lipid oxidation and carbonyl formation were significantly reduced (to 1.35 nmol/mg), heterocyclic amines and poly cyclic aromatic hydrocarbons were reduced by more than 50%, and the flavor was richer, the off-flavor was reduced, and the meat color was bright red for a long time.	[43]
Mutton	Extract	Gas: Synthetic air Minced mutton mixed with 400 ppm nanoencapsulated pomegranate peel extract (PPE) extracted by CP + ultrasound and refrigerated at 4 °C for 12 days.	DBD	Voltage: 120 V, electrode distance: 13 cm, pressure: 2 × 10^−2^ mbar, power: 14 W, processing time: 15 min. Ultrasound: 35% power (275 W), 45 min.	Significantly increased the content of phenols, flavonoids and anthocyanins in pomegranate peel extract, and increased the antioxidant capacity by about 25%. Minced mutton with 400 ppm nanoencapsulated extract performed best in 12 days of refrigeration: the increase in TVN, TBA and pH was significantly inhibited, the total number of colonies was reduced by about 2 log CFU/g, the color and texture were well maintained, and the shelf life was extended by about 6 days, which was better than the free extract and tert-butylhydroquinone (TBHQ).	[44]

## Data Availability

No new data were created or analyzed in this study. Data sharing is not applicable to this article.
